# A Clinical Entity Often Missed—Solitary Rectal Ulcer Syndrome in Children

**DOI:** 10.3389/fped.2020.00396

**Published:** 2020-07-17

**Authors:** P. Thirumal, B. Sumathi, D. Nirmala

**Affiliations:** ^1^Gastroenterology Clinic, Sri Manakula Vinayagar Medical College and Hospital, Puducherry, India; ^2^Gastroenterology, Institute of Child Health and Hospital for Children, Chennai, India

**Keywords:** bleeding per rectum, mucorrhea, constipation, solitary rectal ulcer, children

## Abstract

**Background:** Solitary Rectal Ulcer Syndrome (SRUS) was a relatively uncommon and easily misdiagnosed clinical entity in children. The diagnosis of this condition was often delayed due to lack of clinical suspicion. Only case series were available and no definitive treatment was postulated. Here, we share our experience of SRUS in our institute and reviewed the literature published so far.

**Aim:** To study the clinical profile and treatment response of Solitary Rectal ulcer Syndrome in Children (SRUS).

**Materials:** The clinical profile and 1 year follow up response of the diagnosed cases of SRUS over a period of 5 years was retrospectively collected from medical record department.

**Results:** The median age of presentation among 24 children was 8 years with majority (75%) above 5 years. All children presented with intermittent rectal bleeding with median duration of 5.5 months. The other presenting symptoms documented were hard stool (79%), mucorrhea (70%), and abdominal pain (58%). One child presented with rectal prolapse. On colonoscopy, 46% had single ulcer while another 46% had multiple ulcers and 8% had polypoidal lesion. All lesions were within distal rectum and had characteristic histological pattern. All children were treated with conventional treatment like dietary fibers and laxatives along with toilet training. About 75% children attained remission and 25% had relapse but responded with corticosteroid enema. None required surgery.

**Conclusion:** Conventional treatments itself induce and maintain remission in most of SRUS patients if treatment is instituted at the earliest. Thus, early suspicion and diagnosis is needed to achieve remission.

## Introduction

Bleeding per rectum is one of the common presenting complaints seen in pediatric clinic. The common differential diagnosis are infectious/allergic colitis, colonic polyps, Inflammatory Bowel Disease (IBD), anal fissure and rare conditions like rectal colopathy, vascular ectasia. Solitary rectal ulcer syndrome (SRUS) is often missed at early clinical presentation due to lack of clinical suspicion and usually diagnosed lately.

SRUS is a benign chronic disorder often related to abnormal defecation or straining during defecation. It was well-recognized in the adult population with an incidence of 1 in 100,000 (1) and less common in childhood period. Lack of distinct clinical presentation and varying symptomology, diagnosis is often delayed if not suspected. And since it is a masquerader of IBD and polyps, misdiagnosis may lead to treatment disaster and unwanted surgery. Only few case reports and case series have been reported so far in children. We report a series of 24 children with SRUS and their treatment response.

## Materials and Methods

Retrospective analysis of case records during the period 2012–2017 was done at Department of Pediatric Gastroenterology, Institute of Child Health, Chennai, India. Histologically confirmed SRUS cases were selected and informations such as demographic profile, clinical presentations, routine investigations (complete hemogram, renal function test, stool, and urine routine) and colonoscopic findings were obtained from medical record department. In addition to this, type of treatment, and its response over follow-up period of 1 year were analyzed.

## Results

**Figure 1:**
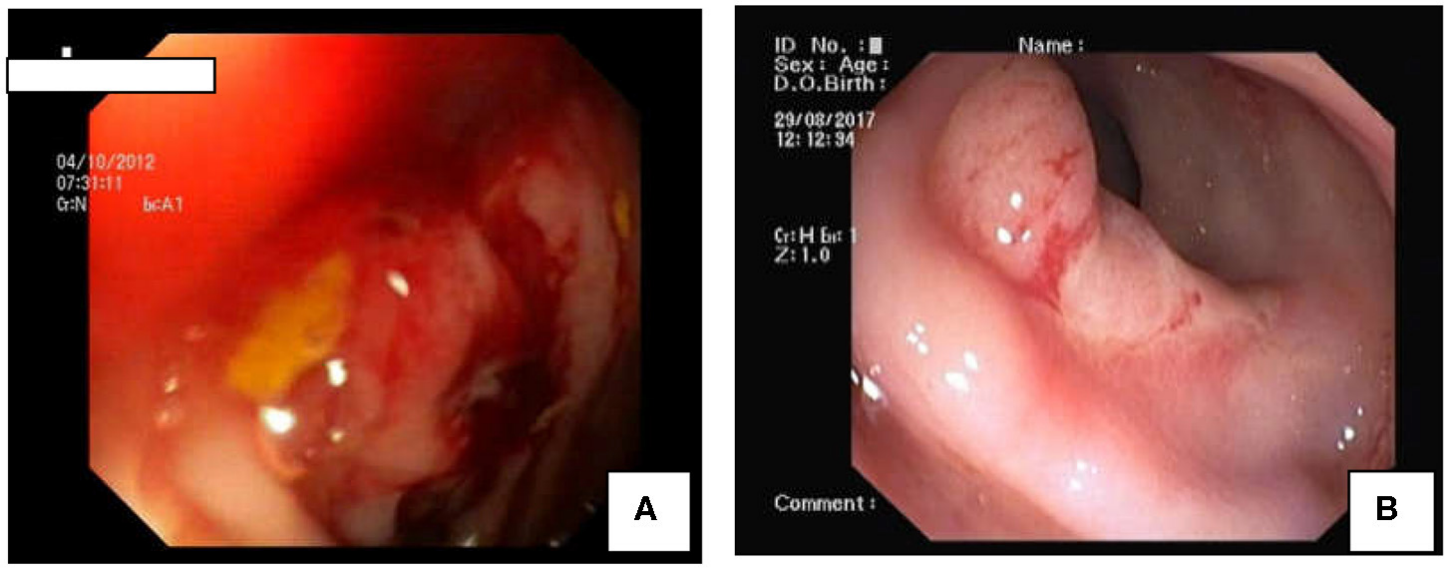
Colonoscopic Findings in SRUS: **(A)** Polypoidal mass like lesion. **(B)** Well-demarcated ulcer in rectum.

**Figure 2:**
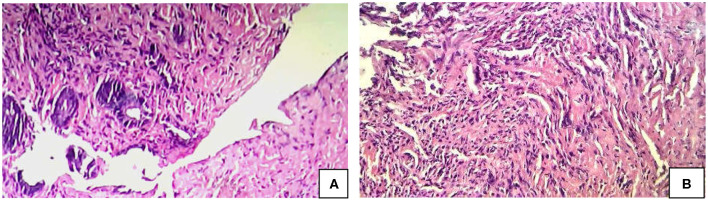
Histology (H & E) Findings in SRUS. **(A)** Complete ulceration of lining mucosa. **(B)** Obliteration of the lamina propria by fibromuscular proliferation of the muscularis mucosa admitting with few inflammatory cell composed of lymphocytes and plasma cells.

Total number of children with SRUS was 24. The median age of presentation was 8 years ranging between 5 and 12 years with Male to Female ratio of 1:1.1. Intermittent rectal bleeding was the presenting complaint in all cases. Mucorrhea (70.8%) and abdominal pain (58.3%) were the associated symptoms. The median duration from onset of symptoms to diagnosis was 5.5 months (IQR 3.0–7.75) with 70 percent of children had symptoms for < 6 months duration. Straining at defecation and passage of hard stool was present in 19 (79.1%) and there was a need for digital evacuation in 27% of children. Four children (16.6%) presented with diarrhea. Rectal prolapse was documented in one child. Anemia (Hb < 10 gm/dl) was most commonly seen in 79.1% of cases. The colonoscopy findings revealed that 11 (45.8%) children had single ulcer in the anterior wall of rectum, another 11 (45.8%) children had multiple ulcers and two children (8.3%) had polypoidal lesion (Table 1). Size of ulcers ranged from 0.5 to 4 cm in diameter but majority were 1–1.5 cms in diameter (Figure 1). All the lesions were in rectum and within 5–10 cms from anal verge. Histology finding documented were presence of fibromuscular obliteration in all cases (100%) followed by surface ulceration with minimal inflammation in 22 (92%) and hypertropic muscularis mucosa with splayed fibers in 21 (87.5%) (Figure 2). A mixed inflammatory infiltrate was also encountered in 3 cases, but cryptitis or crypt abscesses and chronic changes characteristic of IBD were not seen in any biopsies.

**Table 1:** Demographic profile, clinical presentation, colonoscopy findings and histological findings (*N* = 24).

**Characteristics**	**N (%)**
**Demographic profile**	
Median age of presentation	8 years (IQR 5.75–11)
Male: Female ratio	1:1.1
**Clinical presentation**	
Median duration of symptoms	5.5 months (IQR 3.0–7.75)
Rectal Bleeding	24 (100)
Straining during defecation	19 (79.1)
Mucorrhea	17 (70.8)
Abdominal pain	14 (58.3)
Rectal Prolapse	1 (4.2)
**Stool consistency**	
Hard stool	19 (79.1)
Loose stool	4 (16.6)
Digital evacuation	6 (25)
Anemia	19 (79.1)
**Colonoscopy findings**	
Single ulcer	11 (45.8)
Multiple ulcer	11 (45.8)
Polypoidal lesion	2 (8.3)
**Histological findings**	
Fibromuscular obliteration of the lamina propria	24 (100)
Surface ulceration with minimal inflammation	22 (91.6)
Hypertrophic muscularis mucosa with splayed fibers	21 (87.5)
Mixed inflammatory infiltrate with branching and distorted glandular crypts	3 (12.5)
Cryptitis or crypt abscesses and chronic changes	0

*IQR, Inter-Quartile Range*.

All children with constipation were treated with dietary fibers (age in years + 5 g per day), toilet training and followed by laxatives like Polyethylene glycol—PEG 3,350 (0.5–1 g/kg once daily) or Lactulose (1 ml/kg twice daily initially then titrated to achieve 2 soft stools per day) or Liquid paraffin 1 ml/kg/day oral solution for 4–8 weeks. Parents were reassured of the benign nature of the disease and need for regular toilet habits to prevent relapses. About 18 children out of 24 (75%) responded to above treatment and maintained remission till the follow up period of 1 year. Six children (25%) had recurrent rectal bleeding and were treated with corticosteroid rectal enema for 5–7 days. One child had profuse recurrent rectal bleeding despite medical therapy and was referred to higher center for Argon Plasma coagulation. But the child was lost to follow-up. None of our children required surgery (Table 2).

**Table 2:** Treatments and its response (*N* = 24).

**Treatment**	**Response N (%) (Follow-up of 1 year)**
**Conventional treatment**	**(*****N*** **=** **24)**
Toilet Training and High Fiber Diet: ≥ (Age in yrs + 5) gms ± Osmotic Laxatives/Stool softeners	Remission 18 (75) Relapse 6 (25)
{PEG(0.5–1 gm/kg OD) or Lactulose (1 ml/kg BD) or Liquid Paraffin (1 ml/kg OD) per oral}	
**Other treatments**	**(*****N*** **=** **6)**
Rectal Steroid enema	Remission 5 (83.3)
	Failure 1 (16.7)
Argon Plasma Coagulation(1 child)	Lost to follow up (1)

## Discussion

In the year 1829, Cruveilhier (2) had reported four unusual cases of rectal ulcers. Lloyd-Davis used the term “solitary ulcers of the rectum” in the late 1930s. The disease became widely recognized after a review of 68 cases by Madigan et al. (3) Later, Rutter et al. (4) also reported the detailed pathogenic concept of the disease. Solitary rectal ulcer is a misnomer since only 40% of patients had ulcer and others presentations were hyperemic mucosa to broad-based polypoidal lesions. Lesions may be of varying size and shape and ulcer may be solitary or multiple (5) and may involve sigmoid colon also.

High index of clinical suspicion is needed and diagnosis is based on symptomatology in combination with endoscopic and histological findings. The pathophysiology of SRUS is incompletely understood. Inappropriate contraction of the puborectalis muscle, chronic mechanical, and ischemic trauma, inflammation by hard stools and rectal mucosal prolapse have been commonly implicated (6).

In the present study, majority (75%) of children were aged more than 5 years at the time of presentation as comparable with other cases series (7–9) and youngest patient in our study was 1.5 years. Our series observed slight female predominance in contrast to Suresh et al. (7) and Kennedy et al. (10).

Intermittent rectal bleeding and/or mucorrhea were the commonest presentation similar to other reported series (7, 11–15) Predisposing factors for SRUS like constipation (hard stool), straining during defecation were present in majority of case series (7, 12, 14–16). The other presenting symptom which signifies rectal diseases like tenesmus, rectal prolapse were present in varying proportions (7, 12, 14–17). One of the importance and significant observation was that median time interval between onset of index symptoms to diagnosis was 5.5 months in our study series whereas, it was found to be ranging from 6 months to 3.2 years in the other series reviewed (Table 3). The reason for our early diagnosis may be due to early clinical suspicion and evaluation for SRUS earliest at after ruling out common causes. Clinical symptoms in favor of SRUS were features of dyssynergic defecation such as straining during defecation, hard stool, sensation of incomplete evacuation, and digital evacuation.

**Table 3:** Summary of case series reported in literature over past 20 years.

**Case series**	***N***	**Age in years**	**Duration from symptoms to diagnosis**	**Symptoms**	**Colonoscopic finding**	**Treatment given**	**Outcomes**
Godbole et al. (18)	2	Mean: 13 ± 1	2–5 years	RP, RB	U, Po	R	Remission (2)
Kiristioglu et al. (16)	4	NA	NA	M, RB, C, AP	NA	T, L, Su, Sc	Remission (4)
Ertem et al. (6)	2	Mean: 12.5 ± 1.5	2–6 years	RB, RP, C	U	L, Su, St, R	Relapse (2)
Gabra et al. (9)	3	Median: 2.5 (Range: 2–15)	1–2 years	S, RB	U	F, Sur	Remission (2) Relapse (1)
de Carpi et al. (19)	3	Mean: 11 ± 2.1	NA	RB	NA	NA	NA
Somani et al. (20)	24	NA	Mean: 12.6 ± 4.6 months	RB	U	BT, L, APC	Remission (24)
Suresh et al. (7)	22	Median: 10 (1.5–18 y)	Mean: 6 months	RB, M, C, RP	E, U, Po	T, F, Su, M	Remission (14) Relapse (8)
Blackburn et al. (17)	8	Mean: 9.87	Mean: 1.73 yrs (Range:1 m−7 yrs)	S, RB, C, M	U, E	T, L	Remission (4) Relapse (2) Lost to follow up(2)
Perito et al. (12)	15	Median: 13.9 (IQR 9.8–15.6)	Median: 3.2 (IQR 1.2–5.5) yrs	RB, D, C	E, U, Po	L, M	Lost to follow up (6) Response (6) Relapse (3)
Urganci et al. (11)	6	Median:13 (IQR 12–14)	Median: 1 yr (IQR 0.25–4)	C, D, RB, RP	U & Po	Me, St, Sc	Remission (6)
Dehghani et al. (14)	55	Mean: 10.4 ± 3.7	Mean: 15.5 ± 11.2 months	RB, C, M, T, D	E, U, Po	F, Su, Sc, St, R	Lost to follow up (12) Remission (30) Not in remission (13)
Anjum et al. (21)	21	8–12	NA	M, C, T	E, U, Po	NA	NA
Kowalska et al. (13)	31	13 (Range: 5–18)	1–48 months	RB, M, AP	U	T,F, L, Me, Su, Sc, BT, APC, Sur	Response (20) Failure (11)
Podder et al. (15)	140	Median: 12 (IQR 10–14)	Median: 21 (IQR 9–36) months	RB, C, S, D, RP, T	U	T, BT, Local therapy	Lost to follow up (27) Remission (71)
Present study	24	Median: 8 (IQR 5.75–11)	Median: 5.5 (IQR 3–6) months	RB, M, C, RP, D, AP	U, Po	T, L, St	Remission (23) Lost to follow up (1)

*N, number of children; NA, source not available*.

*RB, rectal bleeding; RP, rectal prolapse; M, mucorrhea; S, straining; C, constipation; D, diarrhea; T, tenesmus; AP, abdominal pain*.

*U, ulcer; Po, polypoidal lesion; E, erythema; T, toilet training; F, fiber; L, laxatives; Me, mesalamine tab; St, corticosteroid enema; Sc, sucralfate enema; Su, sulfasalazine enema; Inj.St, injection corticosteroid; R, rectoplexy; BT, bio-feedback training; APC, argon plasma coagulation; Sur, surgery*.

The next work-up was sigmoidoscopy with biopsy to rule out IBD. Endoscopic findings suggestive of SRUS were discrete well-demarcated single or multiple ulcers/erythema in contrast to continuous, symmetrical erythema/hyperemia mucosa with/without ulceration, and loss of vascular pattern in ulcerative colitis. Typical histological findings were fibrous obliteration of the lamina propria, streaming of fibroblasts, and muscle fibers between crypts, thickening or hyperplasia of muscularis mucosa, branching, and distorted glandular crypts, surface ulceration with minimal inflammation (7). Thus, diagnosis was ascertained by combination of clinical symptoms, colonoscopic findings, and histological examination.

Treatment was not standarized and various medicationas and surgical procedures had been tried (22, 23). Response to treatment was not consistent in the reviewed cases series (Table 3). Treatment should include reassurance of the patient and parents that the lesion is benign and chronic. High-fiber diet and appropriate toilet training in young and biofeedback therapy (24) in adolescents had shown encouraging results. Sucralfate enema may be effective in some patients (25) Sulfasalazine (26) and corticosteroids (8, 11) had been tried with varying response. Other treatment modalities include endoscopic application of human fibrin sealant (27), laser therapy (28), argon plasma coagulation (20). In contrast to other case series, we had observed remission in 75% of children with conventional treatment of laxative along with toilet training and only 25% needed rectal corticosteroid enema to achieve remission. Traditional squatting position for defecation and fiber rich staple food intake practices in our region may be a reason for achieving high remission by conventional treatment.

On reviewing case series published over past 20 years (Table 3), the major difference we observed in our present study was that early diagnosis and treatment by conventional methods itself achieve remission and decreases the morbidities due to SRUS in significant proportion.

## Conclusion

We conclude that conventional treatments itself induce and maintain remission in most of SRUS patients if treatment is instituted at the earliest. Hence, early suspicion and diagnosis of SRUS must be considered in a child with bleeding per rectum.

## Data Availability Statement

The raw data supporting the conclusions of this article will be made available by the authors, without undue reservation, to any qualified researcher.

## Author Contributions

PT: data collection, analysis, and manuscript writing. BS: guidance on manuscript writing. DN: revision and final approval. All authors contributed to the article and approved the submitted version.

## Conflict of Interest

The authors declare that the research was conducted in the absence of any commercial or financial relationships that could be construed as a potential conflict of interest.

## References

[1] Martin CJ Parks TG Biggart JD. Solitary rectal ulcer syndrome in Northern Ireland. 1971-1980. Br J Surg. (1981) 68:744–7. 10.1002/bjs.1800681021 7284739

[2] Cruveilhier J Ulcere chronique du rectum. Anatomie Pathologique du Corps Humain. Paris: JB Bailliere (1829). p. 1829–42.

[3] Madigan MR Morson BC. Solitary ulcer of the rectum. Gut. (1969) 10:871–81. 10.1136/gut.10.11.871 5358578 PMC1553062

[4] Rutter KR Riddell RH. The solitary ulcer syndrome of the rectum. Clin Gastroenterol. (1975) 4:505–30. 1183059

[5] Tjandra JJ Fazio VW Church JM Lavery IC Oakley JR Milsom JW. Clinical conundrum of solitary rectal ulcer. Dis Colon Rectum. (1992) 35:227–34. 10.1007/BF02051012 1740066

[6] Ertem D Acar Y Karaa EK Pehlivanoglu E. A rare and often unrecognized cause of hematochezia and tenesmus in childhood: solitary rectal ulcer syndrome. Pediatrics. (2002) 110:e79. 10.1542/peds.110.6.e79 12456946

[7] Suresh N Ganesh R Sathiyasekaran M. Solitary rectal ulcer syndrome: a case series. Indian Pediatr. (2010) 47:1059–61. 10.1007/s13312-010-0177-0 20453265

[8] Dehghani SM Haghighat M Imanieh MH Geramizadeh B. Solitary rectal ulcer syndrome in children: a prospective study of cases from southern Iran. Eur J Gastroenterol Hepatol. (2008) 20:93–5. 10.1097/MEG.0b013e3282f1cbb6 18188027

[9] Gabra HO Roberts JP Variend S Shawis RN. Solitary rectal ulcer syndrome in children. A report of three cases. Eur J Pediatr Surg. (2005) 15:213–6. 10.1055/s-2004-821180 15999319

[10] Kennedy DK Hughes ES Masterton JP. The natural history of benign ulcer of the rectum. Surg Gynecol Obstet. (1977) 144:718–20. 850856

[11] Urganci N Kalyoncu D Eken KG. Solitary rectal ulcer syndrome in children: a report of six cases. Gut Liver. (2013) 7:752–5. 10.5009/gnl.2013.7.6.752 24312719 PMC3848538

[12] Perito ER Mileti E Dalal DH Cho SJ Ferrell LD McCracken M. Solitary rectal ulcer syndrome in children and adolescents. J Pediatr Gastroenterol Nutr. (2012) 54:266–70. 10.1097/MPG.0b013e318240bba5 22094902 PMC3719860

[13] Kowalska-Duplaga K Lazowska-Przeorek I Karolewska-Bochenek K Woynarowski M Czaja-Bulsa G Stawarski A. Solitary rectal ulcer syndrome in children: a case series study. In: Kowalska-Duplaga K, Lazowska-Przeorek I, Karolewska-Bochenek K, Woynarowski M, Czaja-Bulsa G, Stawarski A, Pieczarkowski S, Hapyn E, Jozefczuk J, Korczowski B, Szaflarska-Poplawska A, editors. Clinical Research and Practice. Cham: Springer (2017). p. 105–12. 10.1007/5584_2017_2 28255911

[14] Dehghani SM Bahmanyar M Geramizadeh B Alizadeh A Haghighat M. Solitary rectal ulcer syndrome: is it really a rare condition in children? World J Clin Pediatr. (2016) 5:343–8. 10.5409/wjcp.v5.i3.343 27610352 PMC4978629

[15] Poddar U Yachha SK Krishnani N Kumari N Srivastava A Sarma MS. Solitary rectal ulcer syndrome in children: a report of 140 cases. J Pediatr Gastroenterol Nutr. (2020) 71:29–33. 10.1097/MPG.0000000000002680 32097373

[16] Dehghani SM Haghighat M Imanieh MH Geramizadeh B Solitary rectal ulcer syndrome in children. Turk J Pediatr. (2000) 42:56–60. 10731872

[17] Blackburn C McDermott M Bourke B. Clinical presentation of and outcome for solitary rectal ulcer syndrome in children. J Pediatr Gastroenterol Nutr. (2012) 54:263–5. 10.1097/MPG.0b013e31823014c0 22266488

[18] Godbole P Botterill I Newell SJ Sagar PM Stringer MD. Solitary rectal ulcer syndrome in children. J R Coll Surg Edinb. (2000) 45:411–4. 11153436

[19] de Carpi JM Vilar P Varea V. Solitary rectal ulcer syndrome in childhood: a rare, benign, and probably misdiagnosed cause of rectal bleeding. Report of three cases. Dis Colon Rectum. (2007) 50:534–9. 10.1007/s10350-006-0720-1 17080282

[20] Somani SK Ghosh A Avasthi G Goyal R Gupta P. Healing of a bleeding solitary rectal ulcer with multiple sessions of argon plasma. Gastrointest Endosc. (2010) 71:578–82. 10.1016/j.gie.2009.10.038 20189517

[21] Anjum MN Cheema HA Malik HS Hashmi MA. Clinical spectrum of solitary rectal ulcer in children presenting with per-rectal bleed. J Ayub Med Coll Abbottabad. (2017) 29:74–7. 28712179

[22] Haray PN Morris-Stiff GJ Foster ME. Solitary rectal ulcer syndrome–an underdiagnosed condition. Int J Colorectal Dis. (1997) 12:313–5. 10.1007/s003840050113 9401849

[23] Dehghani SM Malekpour A Haghighat M. Solitary rectal ulcer syndrome in children: a literature review. World J Gastroenterol. (2012) 18:6541–5. 10.3748/wjg.v18.i45.6541 23236227 PMC3516213

[24] Malouf AJ Vaizey CJ Kamm MA. Results of behavioral treatment (biofeedback) for solitary rectal ulcer syndrome. Dis Colon Rectum. (2001) 44:72–6. 10.1007/BF02234824 11805566

[25] Zargar SA Khuroo MS Mahajan R. Sucralfate retention enemas in solitary rectal ulcer. Dis Colon Rectum. (1991) 34:455–7. 10.1007/BF02049928 2036924

[26] Kumar M Puri AS Srivastava R Yachha SK. Solitary rectal ulcer in a child treated with local sulfasalazine. Indian Pediatr. (1994) 31:1553–5. 7875821

[27] Ederle A Bulighin G Orlandi PG Pilati S. Endoscopic application of human fibrin sealant in the treatment of solitary rectal ulcer syndrome. Endoscopy. (1992) 24:736–7. 10.1055/s-2007-1010574 1330505

[28] Rau BK Harikrishnan KM Krishna S. Laser therapy of solitary rectal ulcers: a new concept. Ann Acad Med Singapore. (1994) 23:27–8. 8185265

